# Mixtures of Diethyl Azelate as Novel Nonopioid Modalities for Pain Management

**DOI:** 10.7759/cureus.79960

**Published:** 2025-03-03

**Authors:** Elzbieta Izbicka, Robert T Streeper

**Affiliations:** 1 CEO, New Frontier Labs LLC, San Antonio, USA; 2 CSO, New Frontier Labs LLC, San Antonio, USA

**Keywords:** analgesia, anti-inflammatory, chronic pain, diethyl azelate, drug development, nonopiod, non-steroidal, original article, pain, phospholipase

## Abstract

Introduction

Effective pain management is essential for improving the quality of life. Currently, we have medications to address both mild and severe pain, but there remains a therapeutic gap for pain that is not adequately managed by over-the-counter (OTC) or prescription non-steroidal anti-inflammatory drugs (NSAIDs). Prescription opioids can lead to addiction, respiratory suppression, and even death. OTC pain relief options often lack the potency required to alleviate more intense pain, while stronger treatments, though effective, carry risks of addiction and other adverse effects, limiting their long-term use. This situation underscores the urgent need for safer, nonopiate alternatives. Both musculoskeletal pain and pain from animal toxin envenomation share common mechanisms, including structural changes to the plasma membrane that trigger signaling cascades from membrane-associated phospholipases. Diethyl azelate (DEA), a medium-chain fatty acid ester, represents a new class of NSAIDs that reversibly alter plasma membrane structure and function. DEA mitigates insulin resistance, dyslipidemia, and musculoskeletal pain, and inhibits both exogenous and endogenous phospholipases PLD and PLA2, which are involved in pain signaling. This study aimed to evaluate the analgesic properties of DEA in combination with topical penetration enhancers.

Methodology

Analgesic activities of DEA, dimethyl sulfoxide (DMSO), turpentine, 32 miscellaneous terpenes (including D-limonene, menthol, a and b-pinene), cannabinoid oil, pelargonic acid vanillylamide, and non-prescription analgesics drug controls were examined as single entities and in mixtures in cutaneous mechanical sensitivity (CMS) assays that utilized standardized Von Frey monofilaments (fibers) of variable forces. In addition, DEA, DMSO, and D-limonene were tested as single reagents and mixtures in hemolysis assay in vitro. Inhibition of hemolysis was used as a surrogate endpoint for PLA2 enzymatic activity in bee venom.

Results

Mixtures of DEA and DMSO showed synergy that was most pronounced at equimolar ratios of the components. The maximum duration of sensitivity suppression in CMS assay of 72 h was achieved at 78% DEA and 22% DMSO. Multi-component mixtures of DEA, DMSO, limonene, a-pinene (but not b-pinene), and menthol demonstrated additional enhancement of synergy with DEA at a relatively narrow range of concentrations. Both DMSO and limonene showed bell-shaped dose responses, suggesting that the enhancement of the effects of DEA is not merely due to enhancement of tissue penetration. The activities of multi-component mixtures suggested competition between individual components in certain concentration ranges. The synergy of DEA in mixtures with DMSO and limonene in CMS assays was not observed with related diesters of azelaic acid, diethyl suberate, and diethyl sebacate. In hemolytic assays, DEA, DMSO, and limonene were ineffective as single agents at the examined concentrations, but a specific mixture thereof significantly suppressed hemolysis caused by PLA2.

Conclusion

The findings warrant further development of the mixtures of DEA, DMSO, and select terpenes as novel modalities.

## Introduction

Pain affects one-fifth of the human population at any given time, making effective pain management crucial for improving the quality of life for hundreds of millions of sufferers worldwide. Pain is defined as “an unpleasant sensory and emotional experience associated with, or resembling that associated with, actual or potential tissue damage” [1]. Many pain conditions in humans and in animal models start with a stimulus that perturbs the plasma membrane and initiates a signaling cascade from the membrane-associated phospholipase D2 (PLD2) [2]. Phospholipase A2 (PLA2), another member of the phospholipase family relevant to pain signaling, is ubiquitously expressed in species ranging from bacteria to humans. Exogenous and endogenous PLA2s induce pain and inflammation and in some cases exhibit hemolytic activity. Both PLDs and PLAs are viewed as promising therapeutic targets [3,4].

Topical pain medications currently used for the treatment of muscle and joint pain comprise both anti-inflammatory and analgesic active agents. The anti-inflammatory agents reduce inflammation and the pain that arises from inflammation while analgesics relieve pain but do nothing to treat the underlying cause of the pain. Common over-the-counter medications including aspirin and methyl salicylate (wintergreen oil) are non-steroidal anti-inflammatory drugs (NSAIDs). Other products such as Biofreeze® and Bengay® include menthol, an analgesic that induces a cooling sensation. Capsaicin, an active component of hot peppers, depletes a pain-signaling molecule substance P. Low concentration lidocaine analgesic preparations, now available over the counter, may cause severe burning and dizziness. In general, NSAIDs are safe in short-term use but have modest potency and do not address the underlying causes of the common longer-term pain syndromes such as that caused by arthritis. Arthritis pain arises from tissue destruction and abnormal release of inflammatory signaling molecules, which stimulate pain sensors in the affected tissue and in the brain.

Prescription pain medications include more powerful NSAIDs such as diclofenac sodium gel and solution (Voltaren, Flector Patch, Solaraze, Pennsaid), higher strength lidocaine (over 5%), steroids (dexamethasone, hydrocortisone, prednisone, and others), targeted biological drugs, and opiates. These compounds have side effects that limit their long-term use. Voltaren can cause stomach pain, diarrhea, and nausea. Steroids are linked to increased risk of adrenal involution, infections, weight gain, “moon face,” and elevated blood sugar and blood pressure. Targeted biological drugs include Etanercept (Enbrel) and Adalimumab (Humira). Due to the enhanced risk of cancer, these drugs often bear a “black box” warning from the Food and Drug Administration in the United States. Common opioids, such as morphine, codeine, fentanyl, and oxycodone, are highly addictive. In 2024, opioid addiction affected more than 2.1 million people in the United States [5].

A study on mechanisms of general anesthesia revealed an unexpected link between disruption of the structure and function of plasma membrane lipid rafts and downstream signaling critical for pain management [6]. The best studied lipid rafts are enriched in cholesterol, sphingomyelin such as monosialotetrahexosylganglioside 1 (GM1), and the mechanosensitive enzyme PLD2. Inhalational anesthetics increase the membrane fluidity, disrupt GM1 lipid rafts, and cause translocation of PLD2 to the proximity of TREK1, a mechanically activated potassium channel. Then, PLD2 hydrolyzes phosphatidylcholine to choline and phosphatidic acid (PA), which activates TREK1 and its homolog TRAAK and induces potassium influx, leading to unconsciousness and analgesia [6].

Diethyl azelate (DEA) represents a new class of NSAIDs. DEA shares some functional similarities with NSAIDs but also displays striking differences in the mechanism of action. Like NSAIDs, DEA reduces inflammation and pain by suppressing release of inflammatory cytokines and inhibits PLA2 signaling responsible for pain sensation. Yet, unlike many NSAIDs, DEA is not known to inhibit COX but exerts its anti-inflammatory activity through reversible fluidization of the plasma membrane and subsequent modulation of the inflammatory signaling. We refer to molecules such as DEA as membrane active immunomodulators (MAIMs) that target the cell plasma membrane. MAIMs alter membrane fluidity and shift the position of equilibrium of the innate homeostatic mechanism that regulates membrane fluidity, which we named the adaptive membrane fluidity modulation (AMFM) operating in higher animals that is analogous to homeoviscous adaptation in bacteria [7-9]. DEA has shown activity in seemingly unrelated biological systems; it modulated activities of multiple pathogen-associated molecular pattern receptors with greater effects on membrane-associated versus intracellular receptors and mitigated effects of cholera toxin and anthrax lethal toxin, and was effective in vivo against antibiotic-resistant Staphylococcus aureus and Mycobacterium ulcerans [8,10]. In a clinical study of overweight males, DEA significantly reduced fasting glucose and insulin in subjects with insulin resistance and improved the measures of dyslipidemia [11]. DEA and related azelates inhibit enzymatic and hemolytic activities of PLD in venoms of brown recluse spider, and PLA2 activity in bee and snake venoms, and endogenous PLA2 activity in human urine. We proposed that topical DEA may control pain associated with bites of animals whose venoms contain PLD or PLA2 [9]. DEA is expected to inhibit endogenous membrane-associated PLA2 and the inflammatory response mediated by arachidonic acid released from plasma membrane phospholipids by PLA2. Since DEA disrupts GM1-enriched lipid rafts, pain relief may also result the inhibition of PLD2 signaling by DEA [6].

The aim of this study was to examine the analgesic activity of DEA in combination with other agents. The most extensively studied here, dimethyl sulfoxide (DMSO), is an organosulfur compound that, among other effects, increases the plasma membrane permeation of drugs, DNA, and other molecules. In synthetic membranes, DMSO concentrations of <5% induce gradual thinning of the plasma membrane followed by pore formation and a collapse of the bilayer [12]. DMSO also causes rapid but short-lived relief of arthritic pain [13] and has some utility in wound healing [14]. The oral use of DMSO is currently limited to the treatment of systemic amyloid A amyloidosis, a complication of chronic inflammatory disease [15]. Turpentine has been used as a medication for millennia for the treatment of a variety of illnesses, infections, and injuries. Turpentine made in the United States typically contains a-pinene (75 to 85%), varying amounts of b-pinene (up to 3%), camphene (4 to 15%), limonene (5 to 15%), and low percentages of 3-carene and terpinolene. Even low concentrations of terpenes can boost the activity for both hydrophilic and lipophilic drugs [16]. Terpenes such as limonene and menthol exhibit low cytotoxicity in vivo and improve bioavailability of drugs in animal models and in human skin. Limonene has anti-inflammatory activity when applied to the skin and facilitates wound healing in vivo [17]. Menthol stimulates skin nociceptors, initiates release of vasodilator peptides, and increases skin temperature [16].

This work examined analgesic activities of DEA in combination with DMSO and terpenes. We demonstrated superiority of the mixtures of DEA with DMSO, D-limonene, a-pinene, and menthol in comparison with single components. We also observed unexpected synergies of multicomponent mixtures.

## Materials and methods

Chemicals

Diesters of azelaic acid were synthesized from azelaic acid and an alcohol using acid-catalyzed esterification, followed by fractional distillation to 99% purity determined by gas chromatography-mass spectrometry (GC-MS) as described [8]. Unless stated otherwise, chemicals were sourced from Sigma-Aldrich (St. Louis, MO) and Thermo Fisher Scientific (Waltham, MA). Terpenes were sourced from Vigon International (East Stroudsburg, PA).

Cutaneous mechanical sensitivity assay

A common experimental method to gauge the level of stimulus-evoked skin sensitivity employs a set of graded monofilaments known as von Frey fibers [18]. Cutaneous mechanical sensitivity assay (CMS) uses the monofilaments to test sensory levels and obtain data on the status of diminishing or return in sensibility [19,20]. The tests were conducted at approximately the same time in the morning. A subject was positioned face down and prone on a flat surface, and a circular testing area with a radius of 10 cm was marked on the back of the left and right popliteal area behind the knees. To establish a baseline of minimum force to exert a touch sensation, the areas were randomly touched with a monofilament in a series of 10 measurements (five per each side) using monofilaments of increasing force. Each touch was recorded and the subject reported a touch sensation if experienced. A positive score for a monofilament as was defined as more than six reported incidents of the touch sensation. The baseline levels were established in 20 separate experiments.

The Touch Test Sensory Evaluator kit (North Coast Medical Inc, Morgan Hill, CA) was used. The kit contains 20 monofilaments that exert target forces between 0.25 and 512 millinewtons (mN), corresponding to 0.008 and 300 g. The fibers are coded numerically with the logarithm of the applied force in milligrams as follows: 1.65, 2.36, 2.44, 2.83 (normal sensation), 3.22, 3.61, 3.84 (diminished light touch), 4.08, 4.17, 4.31 (diminished protective sensation) and 4.56, 4.74, 4.93, 5.07, 5.18, 5.46, 5.88, 6.10, 6.45, 6.65 (loss of protective sensation). Notably, logarithmic transformation reduces variance (scatter) across the different treatment groups [18].

Solutions of test articles were prepared in isopropyl alcohol. To test DEA and DMSO at their highest equimolar concentrations of approximately 3M each, stock solutions were prepared neat without the diluent and adjusted for the densities of the two compounds, corresponding to 78% v/v DEA and 22% v/v DMSO. These compounds were evaluated as single reagents and in variable ratios of equimolar stocks of DEA at 78% v/v, 58% v/v, 39% v/v, and 19% v/v and DMSO at 22% v/v, 16% v/v, 11% v/v, and 5% v/v (rounded to the nearest integer). With additional components, respective highest concentrations of DEA and DMSO were 77% and 21%. Other test articles included the following turpentine components and related terpenes (with all compounds tested individually at 2% v/v): amyl alcohol, arnica extract, arnica oil, caryophyllene, bisabolol, a-cedrene, carene delta, caryophyllene acetate, caryophyllene, citronellol, cyclomethylene citronol, decyl acetate, ethyl butyrate, ethyl linalool, hexenol cis-3, kalsec pepper extract, lemon terpenes, lime terpenes, D-limonene (further referred to as limonene), mandarin terpenes, menthol, methyl salicylate, myrcene, nonyl acetate, octyl acetate, orange terpenes, a-pinene and b-pinene, tangerine terpene, a-terpeneol, a-terpinene, and valencene. In addition, we examined cannabinoid oil (30%) and pelargonic acid vanillylamide (PAVA) (0.25% to 4%). Over-the-counter analgesics 4% lidocaine and Voltaren (1.16% diclofenac) were used as positive controls.

Following baseline evaluations, test article solutions were applied in a volume of 0.5 mL per testing area and gently rubbed in for 15 seconds (day 1, time = 0). CMS tests were then performed at the earliest time of 5 minutes (0.1 h) and then at 1, 4, 8, 24, 48, 72, and 96 hours. The assay was completed when the touch sensation returned to the baseline and further referred to as the time to baseline (TBL). Unless specified otherwise, the assays were performed in 10 replicates. The area under the curve (AUC) for the course of each test from t = 0 until return to baseline sensitivity was determined using SigmaPlot version 14.5 (Inpixon, Palo Alto, CA).

Hemolysis assay

The hemolysis assay used human peripheral blood erythrocytes and a bee venom preparation with high levels of PLA2 activity. The assays were performed in triplicate repeats as described [9]. A stock solution of bee venom was diluted in phosphate-buffered saline (PBS) with 0.5% Tween (diluent) to yield the extent of hemolysis comparable to Triton-X used as a positive control. All test articles were also prepared in the same diluent. The reagents were preincubated at room temperature for 5 minutes, and a 30-minute reaction was initiated by the addition of the erythrocyte suspension.

Statistical analysis

Statistical analysis was conducted using Student's t-test; p-values <0.05 were considered significant and p-values <0.005 were considered highly significant.

## Results

Evaluation of DEA and DMSO mixtures in CMS assays

CMS assays in the absence of treatment established that the baseline of sensitivity was achieved using a filament with a bending force of 0.02 grams. Subsequently, DEA and DMSO were tested as single agents and as mixtures. The highest relative molar ratio of 1 corresponded to 78% DEA and 22% DMSO (and actual molarities approximately 3M each). Table 1 shows the effects of DEA and DMSO as single reagents and mixtures on the TBL and AUC.

**Table 1:** Relationship between DEA and DMSO concentrations, AUC, and TBL in cutaneous mechanical sensitivity assays. AUC, area under the curve; DEA, diethyl azelate; DMSO, dimethyl sulfoxide; TBL; time to baseline; TBL-C; corrected time to baseline

DEA (%)	78	78	78	78	78	58	58	58	58	58	39	39	39	39	39	19	19	19	19	19	0
DMSO (%)	22	16	11	5	0	22	16	11	5	0	22	16	11	5	0	22	16	11	5	0	1
AUC	137.13	28.37	9.36	0.60	8.96	11.51	29.01	8.15	25.12	4.52	28.28	8.89	36.00	0.65	0.87	3.24	29.35	0.00	4.56	0.00	0.00
AUC stdev	11.45	0.64	0.82	0.01	0.09	0.46	1.74	0.41	1.03	0.32	1.22	0.80	2.04	0.02	0.02.	0.19	0.79	0.33	0.56	0.33	0.00
TBL (h)	72	24	8	1	4	72	24	24	24	4	24	8	8	1	1	4	24	0.1	4	1	0.1
TBL-C (h)	68	20	4	-3	0	68	20	20	20	0	23	7	7	0	0	3.9	23.9	0	3.9	0.9	0

When DEA or DMSO were tested alone at their highest concentrations of 78% and 22%, the respective values of TBL were 4 hours and 5 minutes. Increasing concentrations of DEA and DMSO did not show a linear relationship with TBL and AUC. The longest TBL of 72 hours was measured at the DEA/DMSO percentage ratios of 78/22 and 58/22. The differences in TBLs between activities of mixtures and single components were highly significant except when TBL was <1. Intra-group comparisons between AUC and TBL showed correlations within 10%.

**Figure 1:**
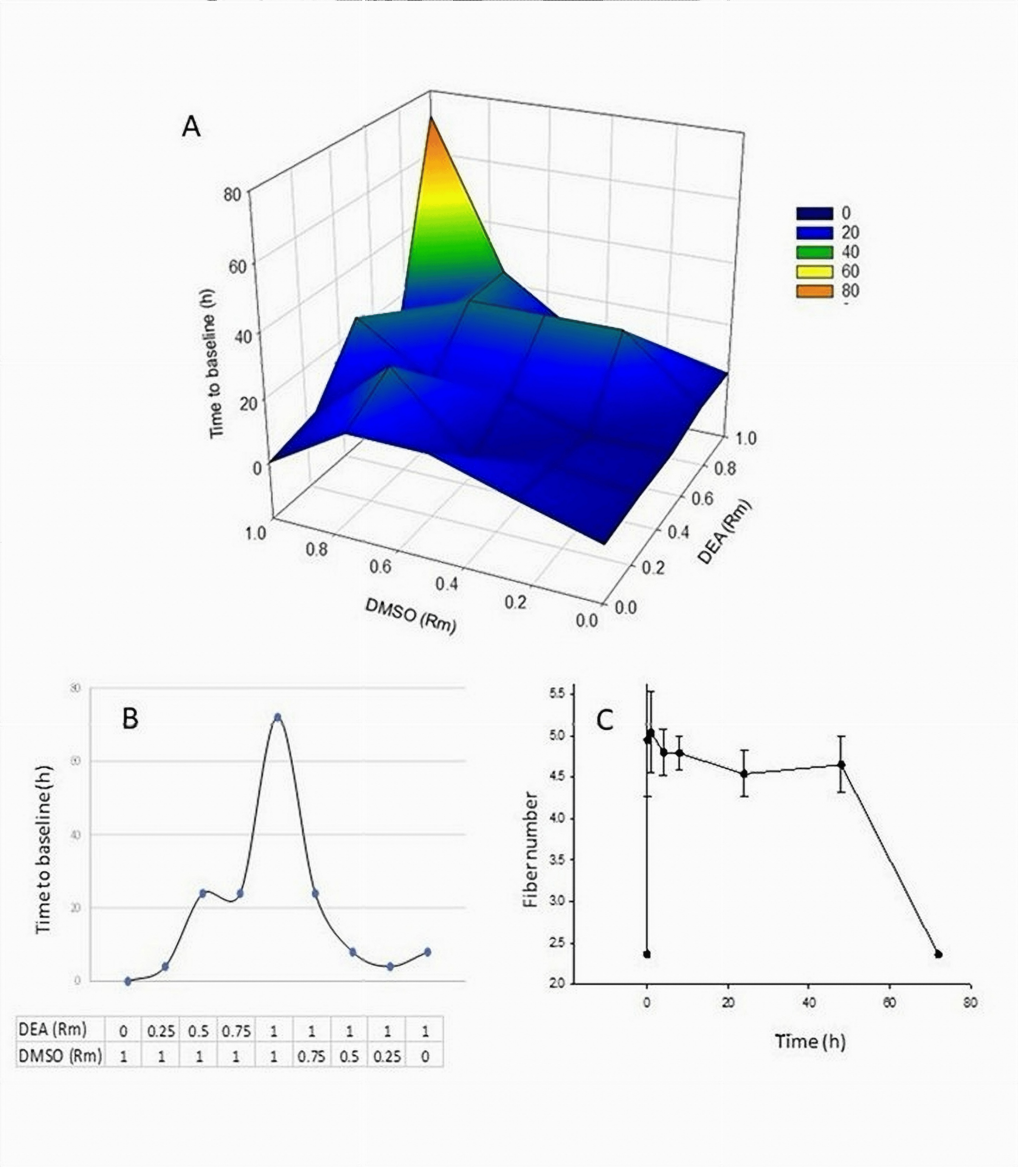
Activities of DEA and DMSO mixtures in cutaneous mechanical sensitivity assays (A) Relationship between relative molarity (Rm) of DEA and DMSO and the time to baseline. The concentration of DEA at Rm = 1 was 78% v/v and that of DMSO at Rm = 1 was 22% v/v. (B): Dose dependence of DMSO in combinations with DEA on the duration of sensitivity suppression (TBL). (C) Time course of DEA and DMSO at 1 Rm each. Error bars indicate means and standard deviations (n=5). DEA, diethyl azelate; DMSO, dimethyl sulfoxide; TBL; time to baseline

The effects of mixture composition on TBL are highlighted in Figure 1A. A non-linear complex relationship between the composition of the mixture and TBL is especially evident at the relative molar ratios above 0.75 of DEA and DMSO each, with a sharp maximum at the 1/1 ratio. A non-linear bell-shaped curve of DMSO and DEA mixture in shown in Figure 1B. DMSO under 0.25 Rm was ineffective, but the analgesic effect steeply increased to a maximum at 1 Rm each (22% DMSO, 78% DEA) and then sharply declined at lower concentrations of DEA (Table 1). These data clearly show that the enhancement of the analgesic effect of DEA is not merely due to enhancement of tissue penetration by DMSO. Figure 1C shows a representative time course of the DEA/DMSO mixture at their highest relative molarities. The curve showed a rapid uptick in suppression of the touch sensation that occurred as early as 5 minutes. A slow decrease over 48 hours was followed by a steep return to the baseline at 72 hours, suggesting a biphasic process. At the peak of sensation suppression at 1 hour, the test fibers applied a bending force of 6-10 g. This corresponded to an approximately 500-fold decrease in cutaneous sensitivity compared to the baseline at a bending force of 0.02 g. Overall, patterns of time course with different combinations of DEA and DMSO exhibited similar trends.

The results demonstrate that certain combinations of DEA and DMSO resulted in synergistic and unexpectedly long duration of touch suppression over time. We refer to this effect as “chronergy.”

Screen of topical analgesics, turpentine, and terpenes in CMS assays

To evaluate activities of topical analgesics as positive controls in our assay system, we examined 4% Lidocaine, Voltaren, and turpentine (also known as terpentine), which is a component of some salves and liniments such as Vicks VapoRub. The TBL values were 4 hours (Lidocaine, turpentine) and 8 hours (Voltaren). Surprisingly, mixtures of 1% or 2% turpentine with 21% DEA and 77% DMSO yielded TBL values ranging from 24 hours to 48 hours relative to the vehicle control.

This finding led us to look closer at turpentine, which is a highly variable mixture. To evaluate the relative contributions of turpentine components and other terpenes, our screens used a mixture of 19% DEA and 5% DMSO and test articles as specified under the Methods section. In all cases, TBL was at least 1 hour for the examined compounds, but the differences between the treatments and control were not statistically significant except for limonene, a-pinene, and menthol. These compounds were further evaluated in multicomponent mixtures with DEA and DMSO.

Activities of mixtures of DEA, DMSO, and limonene in CMS assays

To evaluate the activities of DEA and DMSO mixtures with limonene, we generated a matrix of variable concentrations of the components (Table 2). When tested alone, 2% limonene was inactive in CMS assays. For the combinations of 77% DEA with decreasing concentrations of DMSO (21%, 16%, and 11%) and 2% limonene, the respective TBL values were 72 hours, 24 hours, and 24 hours. However, when the joint contribution of DEA and DMSO was subtracted, the corrected time to baseline (TBL-C) apparently increased from 0 hour to 16 hours. A similar trend was noted at fixed 19% DEA, variable DMSO of 22%, 11%, and 5%, and 2% limonene. Limonene dose response tested in the range of 0.25% to 10% showed a bell-shaped dose response with maximum at 1-2% limonene and TBL-C of 16 hours.

**Table 2:** Relationship between DEA and DMSO concentrations, AUC, and TBL in cutaneous mechanical sensitivity assays. AUC, area under the curve; DEA, diethyl azelate; DMSO, dimethyl sulfoxide; TBL; time to baseline; TBL-C; corrected time to baseline

DEA (%)	DMSO (%)	Limonene (%)	AUC	AUC stdev	TBL (h)	TBL vehicle (h)	TBL-C (h)
77	21	2	137.13	11.45	72	72	0
77	16	2	29.89	3.16	24	24	0
77	11	2	28.52	1.03	24	8	16
58	5	2	29.91	0.79	24	24	0
39	21	2	31.92	0.82	24	24	0
39	11	10	11.51	0.60	8	8	0
39	11	4	14.71	1.18	13	8	5
39	11	2	35.78	1.73	24	8	16
39	11	1	30.68	0.74	24	8	16
39	11	0.25	0.68	0.00	1	8	-7
39	5	2	11.07	0.25	8	1	7
19	21	2	30.52	0.79	24	24	0
19	11	2	10.57	1.36	8	0.1	7.9
19	5	2	35.10	1.60	24	4	20
0	0	2	0.00	0.00	0.1	0	0.1

**Figure 2:**
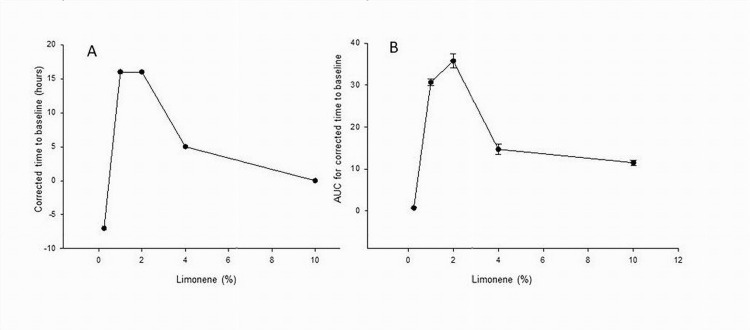
Dose effect of limonene in combination with 20% DEA and 7% DMSO on the corrected time to baseline (A) and AUC (B) in cutaneous mechanical sensitivity assays. AUC, area under the curve; DEA, diethyl azelate; DMSO, dimethyl sulfoxide

We have further examined a dose response of limonene in combination with 20% DEA and 7% DMSO (Figure 2). The peak value of TBL-C of 16 hours was observed with 1% and 2% limonene, but 10% was less effective and 0.25% limonene had no effect. A bell-shaped dose response of limonene with TBL-C as the endpoint (Figure 2A) was mimicked by the AUC curve (Figure 2B).

The dose response of limonene showed similar patterns with a maximum at 1-2% limonene and TBL-C of 16 hours with DEA/DMSO not only at 20%/7% but also at 39%/11%. On the other hand, corresponding DEA/DMSO combinations in the absence of limonene were less effective with respective lower TBL-C of 8 hours and 4 hours. These results suggest possible overlap of different processes with DEA/DMSO affecting plasma membrane fluidity, while the contribution of limonene may happen at a receptor level.

Activities of DEA and DMSO in mixtures with limonene, medium chain fatty acid esters, pinenes, and menthol in CMS assays

**Figure 3:**
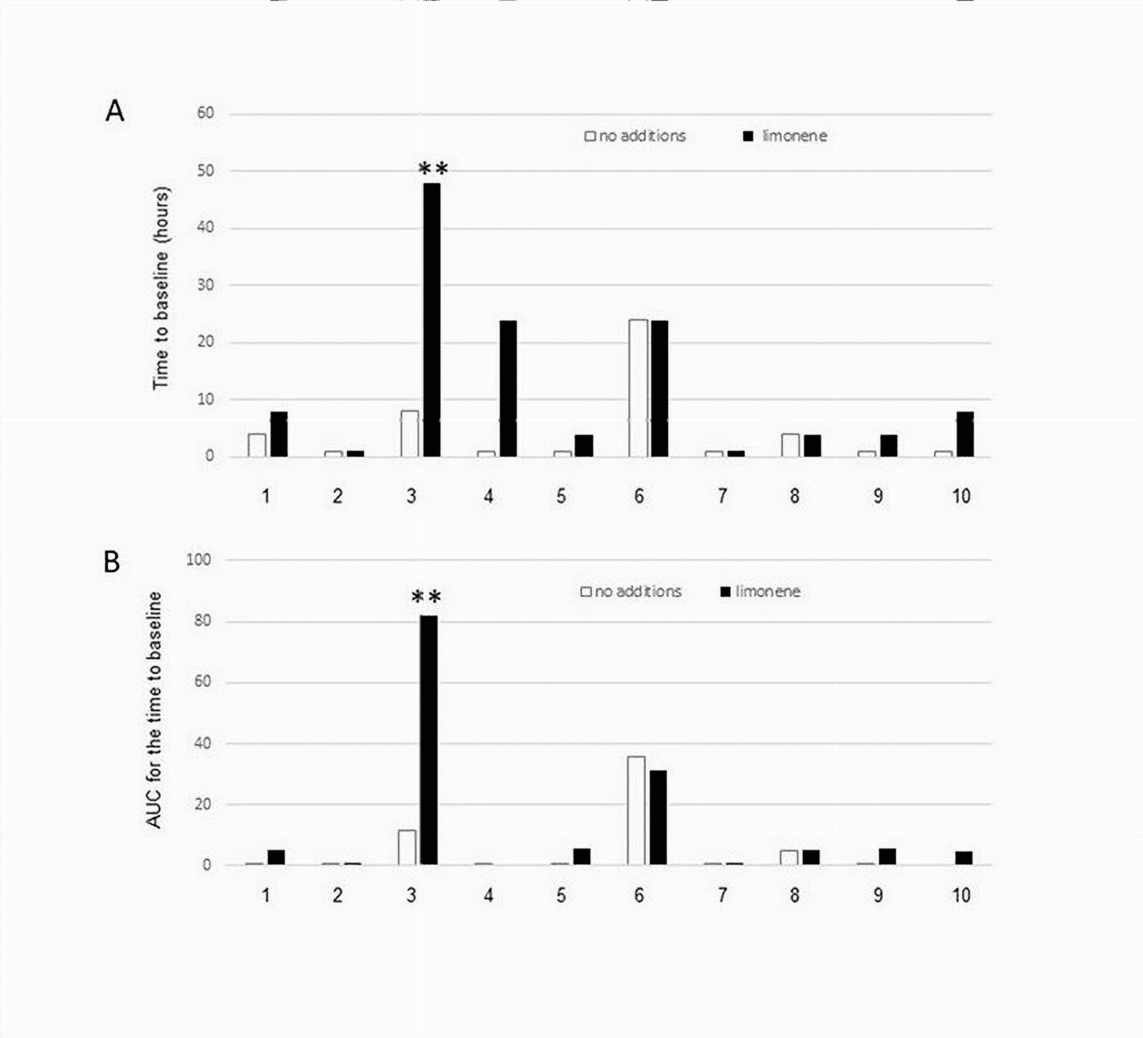
Dose-dependent effects of diesters of medium chain fatty acids (39%) in 11% DMSO alone and in combination with 2% limonene on the duration of sensitivity suppression in cutaneous mechanical sensitivity assays. A, time to baseline; B, AUC. Open bars indicate no addition, and filled bars indicate 2% limonene. 1, diethyl suberate; 2, dimethyl azelate; 3, diethyl azelate; 4, di-2-propyl azelate; 5, di-2-butyl azelate; 6, di-1-pentyl azelate; 7, di-3-pentyl azelate; 8, dicyclohexyl azelate; 9, dioctyl azelate; 10, diethyl sebacate AUC, area under the curve; DMSO, dimethyl sulfoxide

Given the observed synergy between DEA and DMSO that was further enhanced by limonene in CMS assays, we investigated if other azelaic acid esters and related medium chain fatty acid esters shared similar activity. DEA and nine related diesters of azelaic acid, diethyl suberate, and diethyl sebacate were tested at 39% in 11% DMSO in the absence or presence of 2% limonene. DEA was superior to other diesters as measured by TBL (Figure 3A) and AUC (Figure 3B), and in both cases, the activity was further enhanced by limonene. The activity of di-1-pently azelate activity was unaffected by limonene, but the latter significantly enhanced the effect of di-2-propyl azelate.

We subsequently examined pinene isomers in a head-to-head comparison. As Table 3 shows, 2% a-pinene was vastly superior to b-pinene in the mixtures of DEA/DMSO at 39%/11% and 19%/5%.

**Table 3:** Effects of pinene and menthol on the activity of the mixture of DEA, DMSO, and limonene on the duration of sensitivity suppression in cutaneous mechanical sensitivity assays. AUC, area under the curve; DEA, diethyl azelate; DMSO, dimethyl sulfoxide; TBL; time to baseline; TBL-C; corrected time to baseline

DEA (%)	DMSO (%)	Limonene (%)	Pinene (%)	Menthol (%)	AUC	AUC stdev	TBL (h)	TBL-C (h)
39	11	0	2(a)	0	36.00	2.52	24	16
39	11	0	2(b)	0	0.68	0.05	1	-7
39	11	2	2(a)	0	3.87	0.15	4	-4
19	5	0	2(a)	0	33.00	2.17	24	20
39	0	0	0	0.1	0.86	0.08	1	0
39	0	0	0	1	0.73	0.04	1	0
39	0	0	0	2	0.79	0.04	1	0
39	0	0	0	4	0.73	0.04	1	0
39	11	0	0	2	13.07	0.56	8	0
39	11	0	0	0.1	30.71	1.56	24	16
39	11	2	0	1	4.76	0.21	4	-4
39	11	2	0	0.1	72.22	5.85	48	40
19	5	2	0	2	4.82	0.21	4	0
19	5	2	0	0.1	10.91	0.32	8	4

The activity of 2% a-pinene was comparable to that of 2% limonene at the same equimolar DEA/DMSO concentrations. At the same time, the effect of mixture of 2% limonene and 2% b-pinene in DEA/DMSO was unremarkable. Menthol at 0.1% to 4% in mixtures with 39% DEA had a negligible activity, but when 0.1% menthol (but not 2%) was combined with DEA and DMSO, the touch sensation was significantly suppressed over time.

**Figure 4:**
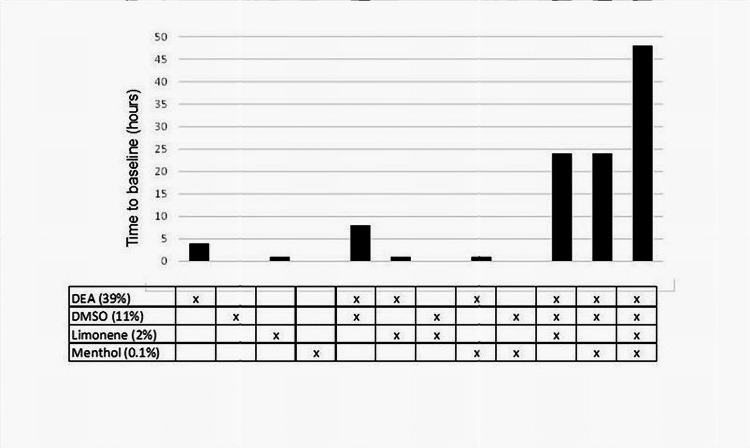
The effects of DEA, DMSO, limonene, and menthol as single agents and in combinations on the duration of sensitivity suppression in cutaneous mechanical sensitivity assays. DEA, diethyl azelate; DMSO, dimethyl sulfoxide

A summary of the key findings from CMS assays (Figure 4) shows the effects of 39% DEA, 11% DMSO, 2% limonene, and 0.1% menthol tested as single agents and in combinations in CMS assays. Note that the concentrations of DEA and DMSO at 0.5 Rm each resulting in TBL of 8 hours were selected to give room for contributions of additional components. Overall, the two mixtures; DEA/DMSO/ limonene and DEA/DMSO/menthol, were both significantly more potent than DEA and DMSO. A four-component mixture of DEA, DMSO, limonene, and menthol had superior activity in comparison to other formulations.

Note that the concentrations of DEA and DMSO at 0.5 Rm each resulting in TBL of 8 hours were selected to allow for contributions of additional components. Overall, the two mixtures, DEA/DMSO/limonene and DEA/DMSO/menthol, were both significantly more potent than DEA and DMSO. A four-component mixture of DEA, DMSO, limonene, and menthol had superior activity in comparison with other formulations.

Evaluation of mixture composition on hemolysis induced by PLA2

**Figure 5:**
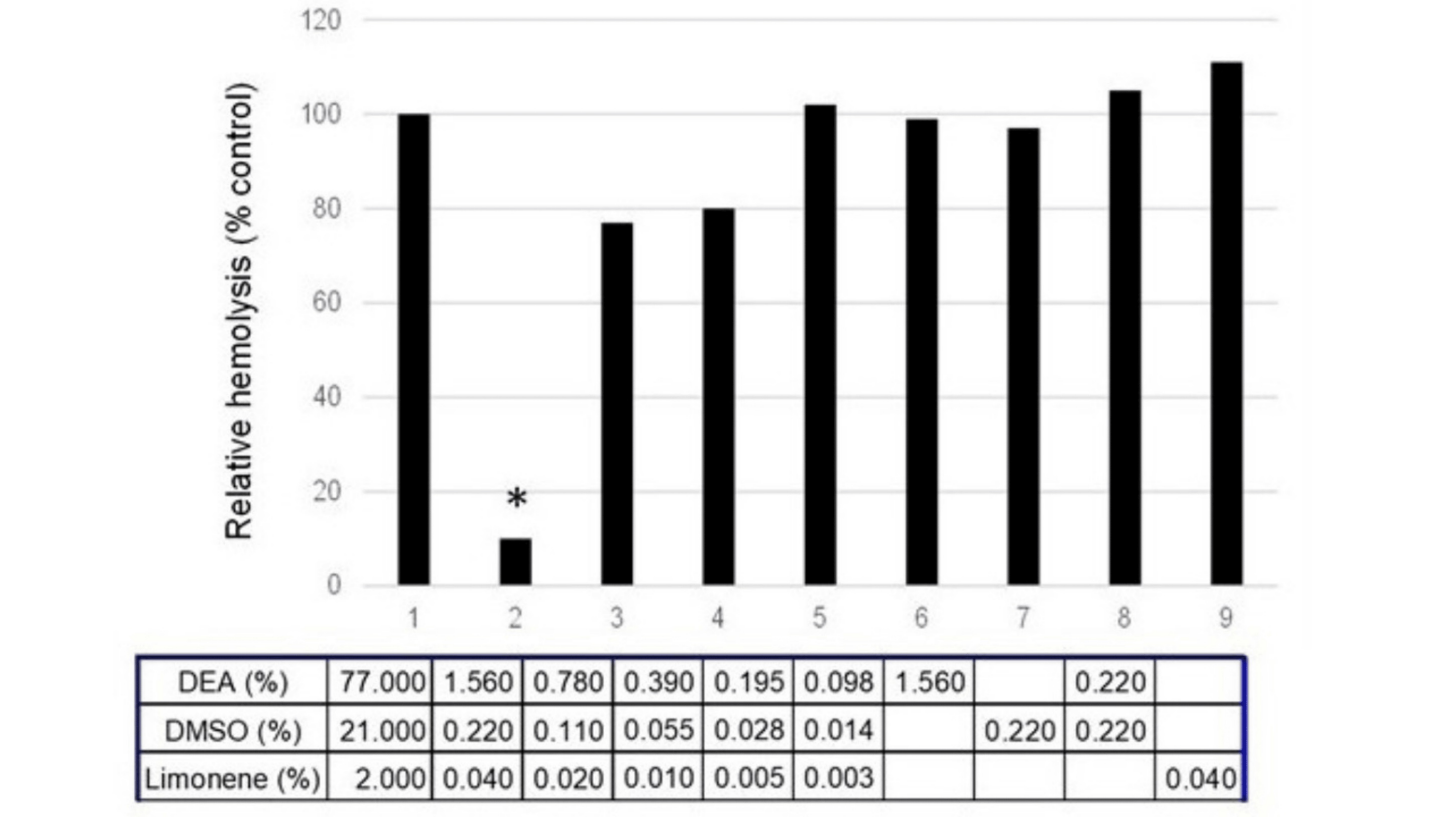
Effect of mixture composition on the hemolytic activity of bee venom. 1, DEA, DMSO, and limonene (77%, 22%, 2%); 2, mixture (1) diluted 50x; 3-5, mixture (2) in 1:2 serial dilutions; 6-9, individual components of mixture (2) at indicated concentrations Control (100%); vehicle alone. DEA, diethyl azelate; DMSO, dimethyl sulfoxide

We have shown before that DEA inhibited PLD in brown recluse spider venom, exogeneous PLA2 activity in bee and snake venoms, and endogenous PLA2 activity in human urine, using blood hemolysis and PLA2 activity as the endpoints [9]. In the present work, we examined the effects of the DEA, DMSO, and D-limonene mixture on hemolysis by bee venom PLA2. Mixtures of DEA, DMSO, and D-limonene mixture (Figures 5; bars 1-5) affected hemolysis with a U-shaped dose response.

The maximum inhibition was seen at 50-fold lower concentrations of these compounds compared to those most effective in CMS tests. At the concentrations corresponding to the maximum inhibition by a given mixture, individual components did not affect hemolysis (bars 6-9).

## Discussion

Our research revealed an unexpected enhancement in the analgesic activity of DEA when combined with DMSO, limonene, α-pinene, and menthol in combinations of two up to four components. Individual compounds had low activities but in combinations exerted unique non-linear dose effects. The first level of synergy was observed in mixtures of DEA and DMSO at relatively high molar concentrations (1M and above), with the maximum effect at equimolar ratios of the components. Another level of synergy occurred when the mixtures of DEA and DMSO were supplemented with limonene, α-pinene, and menthol, all at concentrations lower by at least one order of magnitude than DEA and DMSO. DEA stood out among the examined fatty acid esters as the one that synergized with DMSO and limonene. We also observed the synergy of the mixtures in comparison with single components on the inhibition of human blood hemolysis induced by PLA2.

To the best of our knowledge, this is the first report on multi-level analgesic synergies between any fatty acid ester, DMSO, and terpene. A review of historical data made us realize that the extant reports do not directly apply to our data because they may be limited by the scope or different applications. In the case of DMSO, the compound at 5-15% rapidly blocked conductivity in peripheral nerve C fibers in animal models [21]. In human studies, topical 90% DMSO demonstrated an analgesic effect on nerve conductivity observed within 5 minutes of application [22]. Sparse reports on synergy of DMSO address enhanced activity in combination with antineoplastic agents against human tumors in vitro and in vivo [23]. Analgesic activities of terpenes and terpene synergy have been studied more extensively. Menthol exerts its cooling effect by the activation of sensory neurons known as transient receptor potential (TRP) channels and rapid induction of calcium flux through the channels to induce cold response signals at the application site. Menthol synergistically activates γ-aminobutyric acid (GABA) receptors and sodium ion channels resulting in analgesia [24]. Analgesic activity of limonene is well documented in murine models of chronic musculoskeletal pain, and its mechanism might be also related to GABA and TRPV1 receptors. Synergy of limonene was reported in interactions with multiple antibiotics [25]. Limonene and pinene along with other terpenoids are under investigation for phytocannabinoid-terpenoid interactions that could produce synergy in treatment of pain, inflammation, and other conditions. Inhaled terpenoids can be very potent as they exert activity in vivo and in humans at low ng/ml serum levels, and their therapeutic effects may contribute to the entourage effects of medicinal cannabinoids [26].

DEA together with DMSO and the terpenes, menthol and pinene, are classical examples of MAIMs. Their synergies are likely to arise from complex interactions not only with plasma membranes but also with some integral membrane proteins and membrane-associated proteins. The consequences of the membrane-initiated signaling events taken together with the bell-shaped dose responses are consistent with the MAIM effects relevant for analgesia, further supported by the inhibition of membrane-associated PLA2 [7,9,27].

Pathology of pain has different roots [28]. Nociceptive pain is the most common type of pain, such as pain associated with topical cuts or burns, but also somatic diffuse sensations. It is a physiological protective response to noxious stimuli, and its most representative subtype is inflammatory pain, which can be acute or chronic. Osteoarthritis is a classic example of the latter. Neuropathic pain is a direct consequence of a lesion or disease affecting the somatosensory system, including central neurons and peripheral fibers. Approximately one in 10 people suffer from neuropathic pain, and approximately one in five people suffer from chronic pain. The prevalence of neuropathic pain further increases in individuals with chronic diseases such as diabetic polyneuropathy, cancer, or herpes zoster (shingles).

Analgesic and anti-inflammatory drugs are commonly used in acute and chronic pain management. A large set of Cochrane Reviews comprising 206 studies with more than 30,700 participants assessed the efficacy of topical analgesics applied to intact skin in acute and chronic pain conditions to compare topical analgesics and topical placebo in acute and chronic conditions. In most cases, diclofenac preparations were superior to other treatments. However, in chronic musculoskeletal conditions, topical diclofenac and ketoprofen had limited efficacy in hand and knee osteoarthritis, as did topical high-concentration capsaicin in postherpetic neuralgia. Overall, only a small proportion of people experienced satisfactory pain relief [29]. A novel development in the treatment of knee osteoarthritis came from the observation that the amount of secretory PLA2 (sPLA2) was elevated in the cartilage of mice and humans affected by the disease. To overcome tissue delivery problems, sPLA2 inhibitor was loaded into the phospholipid membrane of engineered micelles, which penetrate deep into the cartilage matrix, had prolonged retention in the joint space, and mitigated progression of osteoarthritis [30].

The mixtures of DEA with DMSO and terpenes may have an advantage over conventional drugs for the treatment of pain, especially chronic pain. DEA alone has a broad spectrum of anti-inflammatory activities and pain modulation through the involvement of PLD and PLA2. The Swiss army knife-like properties of DEA are apparently enhanced in combinations, whereby DMSO might have increased deep tissue penetration of the mixture resulting in unprecedented multi-level synergies.

Limitations

The study evaluated the effects of diethyl azelate mixtures using CMS assay in self-experimentation by a co-author of the manuscript. While the subject was blinded as to the identity of the treatments in the assays and the results were reproducible and statistically significant, this is merely the first report of our observations and we are well aware that our findings need to be independently validated in larger human studies. Interestingly, the synergistic activity of the mixtures was also observed in hemolysis assays, lending support to the observations in skin tests.

Let us keep in mind that self-experimentation in testing drug candidates can be invaluable for researchers. By administering the drug to themselves, researchers gain firsthand insight into the drug's effects, tolerability, and potential side effects. This approach often leads to rapid identification of issues that might not be apparent in preliminary lab tests. It can also foster a deeper understanding of the drug's real-world impacts, enhancing the design of subsequent trials. While it carries inherent risks, self-experimentation can expedite the drug development process and ensure a more thorough assessment of new therapeutic candidates.

## Conclusions

Our study demonstrated that the combinations of DEA and DMSO and select terpenes are significantly more effective than single agents and display multi-level synergies in the mitigation of endpoints relevant for pain. Given the safety profile of DEA and the individual components, the mixtures represent a novel class of nonopioid pain medications with expected favorable safety profiles even in a long-term use. Our findings warrant further development of DEA mixtures for the management of pain and other human ailments.
